# Electrocardiograpic responses during spontaneous hypoglycaemia in people with type 1 diabetes and impaired awareness of hypoglycaemia

**DOI:** 10.1111/dme.70019

**Published:** 2025-02-27

**Authors:** Peter Novodvorsky, Alan Bernjak, Ellen Downs, Amelia Smith, Muhammad Fahad Arshad, Andrei I. Oprescu, Richard M. Jacques, Justin Lee, Simon R. Heller, Ahmed Iqbal

**Affiliations:** ^1^ Clinical Medicine, School of Medicine and Population Health University of Sheffield Sheffield UK; ^2^ Sheffield Teaching Hospitals NHS Foundation Trust Sheffield UK; ^3^ INSIGNEO Institute for In Silico Medicine University of Sheffield Sheffield UK; ^4^ School of Health and Related Research University of Sheffield Sheffield UK

**Keywords:** dead in bed’ syndrome (DiB), electrocardiographic changes, hypoglycaemia, QT‐interval, type 1 diabetes (T1D)

## Abstract

**Aims:**

Hypoglycaemia causes abnormal cardiac repolarisation, which has been related to sympathoadrenal activation. We examined whether individuals with type 1 diabetes (T1D) and impaired awareness of hypoglycaemia (IAH) were protected against proarrhythmogenic alterations on their electrocardiogram during clinical episodes of hypoglycaemia.

**Methods:**

Adults with T1D and IAH underwent 96 h of simultaneous ambulatory electrocardiogram and blinded continuous interstitial glucose (IG) monitoring. Measures of cardiac repolarisation and heart rate variability (HRV) were compared during hypoglycaemia versus time and person‐matched euglycaemia. We compared these data to a historical control group of individuals with T1D and no IAH.

**Results:**

Fourteen individuals (10/14 female) with a mean (SD) age of 39 (10) years and T1D duration of 24 (9) years were examined. Fourteen daytime and 12 nocturnal hypoglycaemic episodes were analysed. During daytime hypoglycaemia versus euglycaemia, the mean (SD) QT_c_ interval was prolonged to 443 (38) versus 422 (27) ms, *p* = 0.027; the Tpeak‐to‐Tend interval was prolonged to 93 (18) versus 77 (9) ms, *p* = 0.002; and the T wave area symmetry decreased to 1.19 (0.37) versus 1.39 (0.23), *p* = 0.014. High‐frequency power decreased during daytime hypoglycaemia versus euglycaemia to 1.66 (0.41) versus 1.92 (0.52), *p* = 0.038. At daytime, the Tpeak‐to‐Tend interval decreased significantly more (hypoglycaemia vs. euglycaemia) in the IAH group in comparison to the decrease observed in the historical control group of T1D individuals without IAH (*p* for interaction 0.005). Cardiac arrhythmias were infrequent and of no clinical significance.

**Conclusions:**

Hypoglycaemia can still lead to proarrhythmogenic electrocardiographic changes in individuals with T1D and IAH. We observed diurnal, inter‐ and intraindividual variability in responses to hypoglycaemia.

**What's new?**

- Hypoglycaemia leads to proarrhythmogenic electrocardiographic changes which are related to sympathoadrenal activation. Individuals with type 1 diabetes (T1D) and impaired awareness of hypoglycaemia (IAH) exhibit impaired sympathoadrenal responses to hypoglycaemia. We aimed to assess whether these individuals were protected against hypoglycaemia induced proarrhythmogenic alterations in their electrocardiogram.
- We showed that hypoglycaemia led to proarrhythmogenic electrocardiographic changes in these individuals, in particular during the day, and these changes were comparable to those observed in individuals with T1D and no confirmed IAH.
- We observed a high degree of inter‐ and intraindividual variability in electrocardiographic responses to episodes of hypoglycaemia

## 1 INTRODUCTION

Over 30 years ago, Tattersall and Gill published the first report of sudden and unexpected nocturnal deaths of 22 individuals with type 1 diabetes (T1D).^1^ Similar reports from other parts of the world followed later.^2^, ^3^ These events were subsequently named ‘dead‐in‐bed’ syndrome (DiB). Autopsies of the deceased could not identify an obvious cause of death, and nocturnal hypoglycaemia has been implicated. This was further substantiated by a publication of a case report describing DiB in a 23‐year‐old person with T1D, in whom a continuous glucose monitoring (CGM) device had confirmed hypoglycemia at the estimated time of death.^4^

Since the original report of DiB, considerable effort has been put into studying this phenomenon with the ultimate goal of preventing these fortunately rare, but tragic and devastating events. Hypoglycaemia has been found to cause proarrhythmogenic abnormalities on resting electrocardiogram (ECG) in experimental and clinical settings independently of the presence or absence of diabetes or the type thereof.^5^, ^6^, ^7^, ^8^, ^9^ Hypoglycaemia leads to prolongation of QT‐interval corrected for heart rate (QT_c_) as well as to changes in rate‐independent indices of cardiac repolarisation, including T‐peak to T‐end interval duration (*T*_p_ *T*_end_) and *T* wave area symmetry ratio (*T*_sym_). Mechanistically, these electrocardiographic changes occur via sympathoadrenal activation, hypokalaemia, and inhibition of rapid delayed rectifier potassium channels (*I*_Kr_).^10^ Of these three mechanisms, sympathoadrenal activation with catecholamine release is the most prominent.^11^ It is therefore possible that malignant cardiac arrhythmias induced by the hypoglycaemia‐driven surge of catecholamines into the circulation may play a causative role in DiB, yet a direct piece of evidence linking cardiac arrhythmias to DiB is still missing.

Impaired awareness of hypoglycaemia (IAH) denotes a diminished ability to perceive acute hypoglycaemia and represents a serious complication of T1D that puts an individual at increased risk of sustaining significant health damage or even death.^12^ Importantly, recurrent hypoglycaemic events progressively impair normal counter‐regulatory responses to hypoglycaemia, such as diminished sympathoadrenal responses, predisposing individuals with IAH to a vicious cycle of ever more frequent hypoglycaemia.^13^, ^14^ Gold and Clarke questionnaires have long been considered the reference standard for the assessment of IAH in both clinical and experimental settings, with a strong correlation between the two questionnaires^15^ Yet, a recently published experimental work suggests a degree of discordance between these questionnaires in the assessment of IAH status.^16^ Importantly, this work also suggested that, at least in a proportion of individuals with T1D, Gold/Clarke questionnaires correlated poorly with adrenaline responses to experimental hypoglycaemia.^16^ Specifically, individuals with partial loss of symptoms or adrenaline responses were more likely to be classified inconsistently when using questionnaires, demonstrating the clinical heterogeneity of IAH.

Given these considerations, we aimed to evaluate if individuals with T1D and IAH would be protected against proarrhythmogenic effects of hypoglycaemia due to their impaired sympathoadrenal responses. To this end, we examined electrocardiographic responses and heart rate variability (HRV) characteristics during spontaneous hypoglycaemia in adults with T1D and IAH and compared these to the responses of individuals with T1D and no confirmed IAH from our previously published study.^9^

## 2 MATERIALS AND METHODS

This was a single‐centre, prospective, observational study. The design of the presented study was similar to previously published observational studies examining electrocardiographic changes during spontaneous hypoglycaemia from our group.^8^, ^9^ Adult individuals with T1D (age ≤55 years, duration of diabetes ≥4 years) and the presence of IAH (defined as Gold score ≥4)^17^ were recruited from Sheffield Teaching Hospitals outpatient clinics between March 2018 and March 2020. Baseline 12‐lead ECG was performed prior to further testing, and participants with bundle branch block or atrial fibrillation were excluded. Individuals with continuous subcutaneous insulin infusion (CSII) devices were allowed to take part in the study, but those with low glucose suspend (LGS) and predicted low glucose suspend (PLGS) features and those on advanced hybrid closed‐loop (AHCL) systems were excluded. Participants with diabetic maculopathy and severe visual impairment, those with myocardial infarction (MI) or coronary artery bypass grafting (CABG) surgery within 12 months prior to inclusion in the study, and participants with estimated glomerular filtration rate (eGFR) <30 mL/min/1.73 m^2^ were also excluded. Data on the presence and severity of diabetes‐related microvascular complications were obtained from the local electronic patient database. This study was conducted in accordance with the ethical standards of the Helsinki Declaration of 1964 and its later amendments. The study protocol was reviewed and received approval by the Yorkshire and The Humber Sheffield Research Ethics Committee, REC reference 17/YH/0365. All participants signed a written informed consent prior to their inclusion in the study.

### 2.1 Baseline Assessment

Anthropometric data and blood pressure were measured, and a venous blood sample was taken to establish creatinine, electrolytes, and glycated haemoglobin A_1c_ (HbA_1c_) at the onset of the monitoring period. HbA_1c_ was established using ion‐exchange high‐performance liquid chromatography. Pregnancy was excluded in all female participants. Cardiovascular autonomic tests were performed in the morning hours at the onset of the monitoring period in accordance with the current consensus statement.^18^ ECG signals were acquired using a g.USBamp biosignal amplifier unit together with the g.Recorder software (g.tec Medical Engineering GMBH, Schiedlberg, Austria) and analysis was undertaken using custom software written in MATLAB (MathWorks, Natick, Massachusetts, USA). Participants were instructed to avoid vigorous exercise, caffeine, and smoking 12 h prior to morning testing. None of the participants were shift workers, and they had a normal day/night sleep cycle. Participants were classified as possible cardiac autonomic neuropathy (CAN) when one cardioreflex test was below the age‐adjusted reference range and definite CAN when two or more cardioreflex tests were below the age‐adjusted reference range.^18^, ^19^

### 2.2 Monitoring

All participants underwent 96 h of simultaneous 12‐lead Holter ECG and blinded continuous glucose monitoring (CGM) whilst carrying on with their usual daily activities and diabetes management. Participants who had their own real‐time CGM device were allowed to use it freely throughout the monitoring period. Twelve‐lead high‐frequency ambulatory ECGs (Mortara H12+; Mortara Instrument Inc. Milwaukee, Wisconsin, USA) were recorded at a 1000 Hz sampling rate with electrodes in a Mason‐Likar configuration. Participants also had a time‐synchronised CGM system attached (Dexcom G6; Dexcom Inc., San Diego, California, USA) in a blinded mode. Participants were asked to keep a record of any symptomatic hypoglycemia in the provided diaries. Any hypoglycemia episode, as defined in the next paragraph, without simultaneous self‐report of symptoms in the diary was regarded as asymptomatic.

### 2.3 CGM Analysis

In this study, hypoglycemia was defined as IG ≤3.5 mmol/L and euglycaemia was defined as an IG between 5 and 10 mmol/L in line with previously published studies.^8^, ^9^, ^20^ We defined a valid hypoglycemic episode as a period of IG below the threshold for ≥20 min.^21^ The first reading of IG ≤3.5 mmol/L marked the start of the hypoglycemia, and the first reading of IG >3.5 mmol/L signified the end of the episode. The lowest IG within the hypoglycemic episode was designated the hypoglycemia nadir and was matched with a euglycaemic time point from the same individual at the same time (within 20 min) on a different day.

### 2.4 Heart Rate Variability Analysis

Indices of heart rate variability (HRV) were calculated from 5‐min segments of R‐R intervals as described previously.^9^ They included time domain indices (standard deviation of normal‐to‐normal intervals, SDNN and root mean square of successive differences, RMSSD), and frequency domain indices (spectral power within the high‐frequency band (0.15–0.4 Hz), log HF, spectral power within the low‐frequency band (0.04–0.15 Hz), log LF, total power, log TotPower, and LH/HF ratio). Log HF reflects parasympathetic activity, and log LF indicates the level of sympathetic modulation in HRV.^22^

### 2.5 Repolarisation Analysis

Repolarisation analysis and detection of QT interval duration were undertaken using custom‐built software as described previously.^9^ QT intervals were corrected for heart rate (QTc) using Bazett's formula.^23^ Cardiac repolarisation was characterised by calculating rate‐independent parameters, including T‐peak to T‐end interval duration (TpTend) and T wave area symmetry ratio (Tsym).^24^ All parameters, including HRV indices, were compared at hypoglycaemia nadir versus time‐ and person‐matched euglycaemia, indicating that only those hypoglycaemic episodes that had time‐ and person‐matched euglycaemia data available were included in the analysis.

### 2.6 Historical control group

A historical control group of individuals with T1D and without confirmed IAH from our previously published work^9^ was used to compare HRV and cardiac repolarisation characteristics with the studied population with IAH. This historical control group included 48 episodes of daytime hypoglycaemia (recorded in 21 individuals) and 41 episodes of nocturnal hypoglycaemia (recorded in 19 individuals) to which person‐ and time‐matched euglycaemia were available. Similar equipment and statistical approaches were used to record the electrocardiogram in both groups.

### 2.7 Arrhythmia Analysis

The 12‐lead Holter ECG data were reviewed using H‐Scribe 4.34 software (Mortara Instrument Inc.). The software automatically detected arrhythmic events according to predetermined event definitions. These included atrial ectopic beats (AEB) (prematurity threshold 30%), bradycardia (consecutive beats at rate <45 bpm for >5 s), single ventricular premature beats (VPBs), complex VPBs (VPB couplets and runs), and total VPBs (sum of all VPBs). Hypoglycaemic hours (mean hourly IG ≤3.5 mmol/L) and euglycaemic hours (mean hourly IG 5–10 mmol/L) were identified for further statistical analysis. Incidence rate differences (IRD) for distinct types of arrhythmias were calculated in participants in whom at least one hypoglycaemic and one euglycaemic hour were recorded. All identified arrhythmic events were manually verified for accuracy and modified if needed. Investigators were blinded to glucose values during arrhythmia identification.

### 2.8 Statistical Analysis

This was an observational study, and thus no power calculations were performed. Duration and nadir values of nocturnal and daytime hypoglycaemic episodes were compared using linear mixed effects models with a random intercept for individuals to allow for repeated measurements per individual. Three‐level linear mixed effects models were used for the analysis of cardiac repolarisation and HRV with random intercepts for pairs of measurements nested within individuals and a fixed factor to compare hypoglycaemia to euglycaemia. Models making comparisons to historical controls included an additional fixed factor comparing individuals with IAH and historical controls, and an interaction with hypoglycaemia status. Data were summarised using mean (SD) except for the duration of hypoglycaemic episodes which was log10 transformed after inspection of residuals and reported as geometric mean (95% CI). Pearson correlations were estimated between electrocardiographic responses to hypoglycaemia and the duration and nadir values of hypoglycaemic episodes. Statistical analysis was performed with R (version 4.2.1; https://cran.r‐project.org/). Linear mixed effects models were fit by restricted maximum likelihood using the nlme package.^25^ A *p* value ≤0.05 was deemed statistically significant.

## 3 RESULTS

### 3.1 Baseline characteristics

In total, 14 individuals were included in the study. Baseline participant characteristics are shown in Table 1. Detailed individual characteristics of all participants, including their insulin regimen, diabetes‐related complications status, and the list of QT‐prolonging medications are shown in Table S1.

**TABLE 1:** Baseline participant characteristics.

Number of participants, *n*	14
Female, *n* (%)	10 (71%)
Age (years)	38.6 ± 9.7 (26–55)
Ethnicity, *n* (%)	
White British	13 (92.9%)
Other	1 (7.1%)
Duration of diabetes (years)	24.0 ± 9.1 (11–42)
BMI (kg/m^2^)	26.0 ± 4.3 (20.1–32.7)
HbA_1C_	
%	9.0 ± 2.1 (6.6–13.1)
mmol/mol	75 ± 23 (49–120)
Systolic BP (mmHg)	118 ± 14 (97–120)
Diastolic BP (mmHg)	68 ± 9 (55–82)
Heart rate (bpm)	78 ± 13 (55–98)
Baseline QTc (ms)	436 ± 12 (416–461)
Potassium (mmol/L)	4.7 ± 0.4 (3.9–5.6)
Creatinine (μmol/L)	68 ± 15 (45–90)
Gold score, *n* (%)	
4	4 (29%)
5	4 (29%)
6	3 (21%)
7	3 (21%)
CAN status, *n* (%)	
No CAN	7 (50.0%)
Possible CAN	3 (21.4%)
Definite CAN	3 (21.4%)
Not determined	1 (7.1%)

*Note*: Data are displayed as mean ± SD (range) or *n* (%).

Abbreviations: BMI, body mass index; BP, blood pressure; bpm, beats per minute; CAN, cardiovascular autonomic neuropathy; QTc, QT interval corrected for heart rate.

### 3.2 CGM data and hypoglycaemia episodes

**FIGURE 1:**
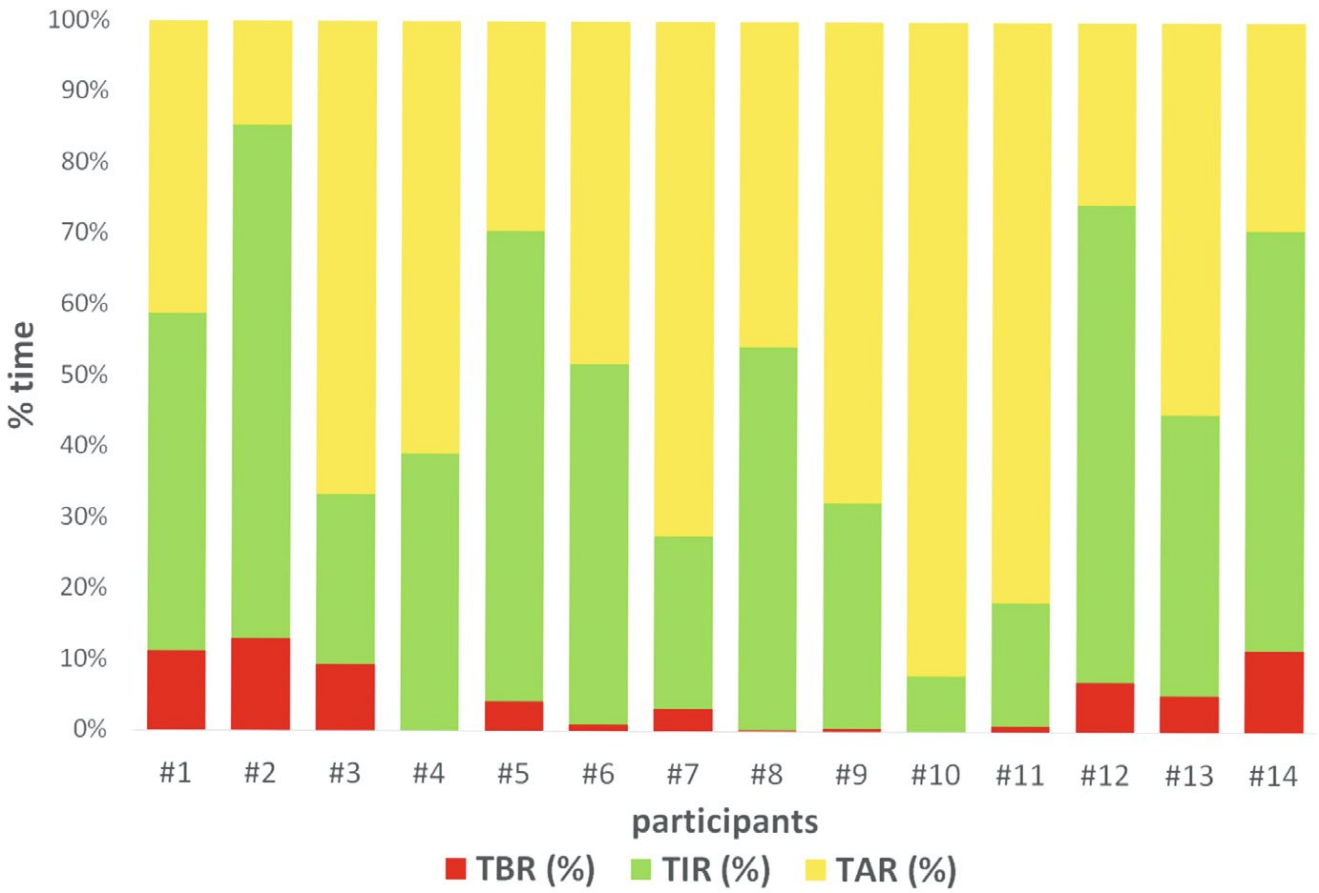
Individual proportions of time spent in defined IG ranges during the 96‐hour‐long monitoring period. TBR, time below range (IG <3.9 mmol/L); TIR, time in range (IG 3.9–10 mmol/L); TAR, time above range (IG >10 mmol/L).

A total of 1214.5 h of CGM data were recorded. Individual proportions of time spent in defined IG ranges^26^ are shown in Figure 1.

During the study period, 8 participants experienced 35 hypoglycaemic episodes, of which 27 (77%) were asymptomatic. Twenty‐two (63%) episodes occurred during the day (17/22, 77% were asymptomatic) and 13 (37%) episodes occurred during the night (10/13, 77% were asymptomatic). The number of hypoglycaemic episodes experienced by each participant is listed in Table S1.

Mean (95% CI) duration of nocturnal hypoglycaemic episodes 82 (50–135) min was longer than the mean (95% CI) duration of daytime hypoglycaemic episodes 46 (46–58) min (*p* = 0.019). Mean (SD) nadir of nocturnal hypoglycaemic episodes 2.89 (0.46) mmol/L did not differ from the mean (SD) nadir of daytime hypoglycaemic episodes 2.73 (0.35) mmol/L (*p* = 0.438).

### 3.3 Electrocardiographic responses, heart rate variability (HRV) and cardiac arrhythmias

In relation to electrocardiographic responses and HRV characteristics, 26 hypoglycaemic episodes (14 daytime and 12 nocturnal episodes) were analysed. Episodes to which at least one person‐ and time‐matched euglycaemic episode could not be found were not included in the analysis. The number of hypoglycaemic episodes experienced by each participant that were included in this analysis is listed in Table S1. In this group of individuals with IAH, the mean QT_c_ interval was prolonged, the mean Tpeak‐to‐Tend interval was prolonged, the T wave area symmetry ratio decreased, and high‐frequency power decreased during daytime hypoglycaemia versus euglycaemia (Table 2). No changes in these responses were observed during nocturnal hypoglycaemia versus nocturnal euglycaemia (Table 3). Next, we compared HRV and electrocardiographic responses to hypoglycaemia with the responses to hypoglycaemia in individuals with no evidence of IAH from our previously published study.^9^ This historical control included 48 episodes of daytime hypoglycaemia (recorded in 21 individuals) and 41 episodes of nocturnal hypoglycaemia (recorded in 19 individuals). At daytime, the Tpeak‐to‐Tend interval duration decreased significantly more (hypoglycaemia vs. euglycaemia) in the IAH group in comparison to the decrease observed in the group of T1D individuals without IAH (*p* for interaction 0.005) (Table 2, last column). No other statistically significant differences in HRV and electrocardiographic responses to hypoglycaemia were noted between these two groups (Tables 2 and 3). We observed a high degree of inter‐ and intraindividual heterogeneity in responses to episodes of hypoglycaemia (Table S2.). Very few cardiac arrhythmias were detected during the 96‐hour‐long study period. These were limited to atrial ectopic beats and single ventricular premature beats and were without clinical significance (Table S3). Last, we correlated QT_c_, Tpeak‐to‐Tend interval duration, and T wave area symmetry ratio with nadir glucose values (Table 4) and the duration of hypoglycaemic episodes (Table 5) together, and separately for day and night. Of these correlations, the strongest one was a positive correlation between hypoglycaemia nadir and T wave symmetry ratio at night (Pearson's *R* 0.86, *p* < 0.001) (Table 4).

**TABLE 2:** Electrocardiographic responses and HRV characteristics of daytime hypoglycaemia episodes.

	IAH group					Historical control					Interaction, *p*‐value
	Participants (episodes)	Hypo	Eu	Mean paired difference (95% CI)	*p*‐Value	Participants (episodes)	Hypo	Eu	Mean paired difference (95% CI)	*p*‐Value	
HR (bpm)	7 (14)	87.6 (16.4)	85.6 (12.5)	2.00 (−4.23 to 8.23)	0.523	21 (48)	82.2 (15.0)	77.9 (12.4)	4.35 (0.99, 7.71)	0.012	0.509
SDNN (ms)	6 (10)	35.7 (12.7)	52.1 (22.8)	−16.5 (−29.7 to −3.3)	0.016	21 (48)	55.8 (21.4)	58.4 (17.4)	−2.7 (−8.7 to 3.3)	0.376	0.062
RMSSD (ms)	6 (10)	12.5 (5.4)	17.2 (9.1)	−4.7 (−10.6 to 1.1)	0.110	21 (48)	25.1 (12.4)	27.7 (13.1)	−2.6 (−5.3 to 0.1)	0.058	0.504
Log LF	6 (10)	2.30 (0.44)	2.47 (0.28)	−0.17 (−0.35 to 0.02)	0.079	21 (48)	2.77 (0.30)	2.84 (0.25)	−0.07 (−0.16 to 0.01)	0.084	0.373
Log HF	6 (10)	1.66 (0.41)	1.92 (0.52)	−0.25 (−0.49 to −0.02)	0.035	21 (48)	2.08 (0.45)	2.17 (0.42)	−0.08 (−0.19 to 0.02)	0.123	0.194
Log (TotPower)	6 (10)	2.40 (0.43)	2.59 (0.33)	−0.20 (−0.38 to −0.02)	0.034	21 (48)	2.87 (0.30)	2.94 (0.27)	−0.07 (−0.15 to 0.02)	0.111	0.198
LF/HF ratio	6 (10)	4.68 (1.92)	4.57 (3.69)	0.11 (−2.38 to 2.60)	0.931	21 (48)	6.46 (5.09)	5.73 (4.24)	0.73 (−0.40 to 1.87)	0.202	0.650
QTc (ms)	7 (14)	443.1 (37.6)	421.8 (26.6)	21.3 (8.5 to 34.0)	0.001	21 (48)	408.0 (27.7)	395.4 (23.9)	12.6 (5.7 to 19.5)	0.001	0.236
TpTend (ms)	7 (14)	93.1 (18.1)	76.4 (8.7)	16.8 (10.2 to 23.3)	<0.001	21 (48)	72.7 (12.6)	66.9 (10.8)	5.8 (2.2 to 9.3)	0.002	**0.005**
Tsym	7 (14)	1.19 (0.37)	1.39 (0.23)	−0.20 (−0.36 to −0.04)	0.017	21 (48)	1.44 (0.38)	1.57 (0.34)	−0.13 (−0.22 to −0.05)	0.003	0.483

*Note*: Data are displayed as mean (SD). Statistically significant comparisons between the IAH group and the historical control group are highlighted in bold (interaction *p*‐value).

Abbreviations: Eu, euglycaemia; HR, heart rate; Hypo, hypoglycaemia; Log (TotPower), total power of HRV; Log HF, high‐frequency power of HRV; log LF, low‐frequency power of HRV; QTc, QT interval corrected for heart rate; RMSSD, root mean square of successive differences; SDNN, standard deviation of normal‐to‐normal intervals; TpTend, T peak to T end interval duration; Tsym, T wave area symmetry ratio.

**TABLE 3:** Electrocardiographic responses and HRV characteristics of nocturnal hypoglycaemia episodes.

	IAH group					Historical control					Interaction, *p*‐value
	Participants (episodes)	Hypo	Eu	Mean paired difference (95% CI)	*p*‐Value	Participants (episodes)	Hypo	Eu	Mean paired difference (95% CI)	*p*‐Value	
HR (bpm)	6 (12)	73.7 (10.7)	72.2 (13.9)	1.50 (−3.76 to 6.75)	0.569	19 (41)	68.0 (12.5)	66.4 (9.1)	1.69 (−1.15 to 4.53)	0.238	0.949
SDNN (ms)	5 (9)	40.6 (24.9)	35.8 (22.1)	4.8 (−22.0 to 31.6)	0.721	19 (41)	67.0 (42.9)	67.3 (34.6)	−0.2 (−12.8 to 12.3)	0.969	0.734
RMSSD (ms)	5 (9)	25.4 (14.0)	20.8 (12.3)	4.7 (−2.3 to 11.6)	0.186	19 (41)	32.8 (15.2)	32.6 (14.3)	0.2 (−3.1 to 3.5)	0.906	0.250
Log LF	5 (9)	2.41 (0.61)	2.27 (0.65)	0.13 (−0.10 to 0.37)	0.260	19 (41)	2.72 (0.51)	2.72 (0.44)	−0.002 (−0.11 to 0.11)	0.973	0.300
Log HF	5 (9)	1.89 (0.55)	1.90 (0.47)	−0.001 (−0.22 to 0.22)	0.993	19 (41)	2.24 (0.49)	2.23 (0.50)	0.01 (−0.10 to 0.11)	0.869	0.938
Log (TotPower)	5 (9)	2.56 (0.55)	2.47 (0.53)	0.09 (−0.13 to 0.30)	0.420	19 (41)	2.87 (0.48)	2.86 (0.43)	0.004 (−0.10 to 0.10)	0.939	0.485
LF/HF ratio	5 (9)	4.92 (4.43)	3.69 (3.28)	1.22 (−0.94 to 3.39)	0.261	19 (41)	3.97 (3.28)	4.11 (3.90)	−0.14 (−1.16 to 0.87)	0.777	0.255
QTc (ms)	6 (12)	424.5 (39.3)	418.5 (31.4)	6.1 (−3.6 to 15.8)	0.215	19 (41)	403.4 (27.0)	397.2 (20.9)	6.2 (0.9 to 11.4)	0.022	0.984
TpTend (ms)	6 (12)	84.7 (19.9)	78.4 (11.3)	6.3 (1.0 to 11.6)	0.021	19 (41)	70.7 (9.3)	66.3 (7.3)	4.4 (1.6 to 7.3)	0.003	0.540
Tsym	6 (12)	1.40 (0.38)	1.51 (0.39)	−0.11 (−0.29 to 0.08)	0.259	19 (41)	1.68 (0.40)	1.79 (0.27)	−0.12 (−0.22 to −0.02)	0.025	0.923

*Note*: Data are displayed as mean (SD).

Abbreviations: Eu, euglycaemia; HR, heart rate; Hypo, hypoglycaemia; Log (TotPower), total power of HRV; Log HF, high‐frequency power of HRV; log LF, low‐frequency power of HRV; QTc, QT interval corrected for heart rate; RMSSD, root mean square of successive differences; SDNN, standard deviation of normal‐to‐normal intervals; TpTend, T peak to T end interval duration; Tsym, T wave area symmetry ratio.

**TABLE 4:** Correlations of electrocardiographic responses to hypoglycaemia with nadir glucose values of hypoglycaemic episodes.

	Total			Day			Night		
	*N*	Pearson's *R* (95% CI)	*p*‐Value	*N*	Pearson's *R* (95% CI)	*p*‐Value	*N*	Pearson's *R* (95% CI)	*p*‐Value
QTc (ms)	26	−0.01 (−0.39 to 0.38)	0.980	14	−0.02 (−0.54 to 0.52)	0.954	12	0.07 (−0.52 to 0.62)	0.819
TpTend (ms)	26	−0.39 (−0.68 to −0.01)	0.046	14	−0.13 (−0.62 to 0.43)	0.646	12	−0.57 (−0.86 to 0.01)	0.053
Tsym	26	0.38 (−0.01 to 0.67)	0.060	14	−0.22 (−0.67 to 0.35)	0.449	12	0.86 (0.57 to 0.96)	<0.001

Abbreviations: QTc, QT‐interval corrected for heart rate; TpTend, T‐peak to T‐end interval duration; Tsym, T wave area symmetry ratio.

**TABLE 5:** Correlations of electrocardiographic responses to hypoglycaemia with the duration of hypoglycaemic episodes.

	Total			Day			Night		
	*N*	Pearson's *R* (95% CI)	*p*‐Value	*N*	Pearson's *R* (95% CI)	*p*‐Value	*N*	Pearson's *R* (95% CI)	*p*‐Value
QTc (ms)	26	−0.01 (−0.39 to 0.38)	0.976	14	0.30 (−0.28 to 0.72)	0.300	12	−0.08 (−0.62 to 0.52)	0.813
TpTend (ms)	26	0.37 (−0.02 to 0.66)	0.062	14	0.52 (−0.01 to 0.82)	0.056	12	0.44 (−0.17 to 0.81)	0.148
Tsym	26	−0.29 (−0.61 to 0.11)	0.150	14	0.04 (−0.50 to 0.56)	0.892	12	−0.68 (−0.90 to −0.18)	**0.015**

*Note*: Statistically significant correlations are highlighted in bold.

Abbreviations: QTc, QT‐interval corrected for heart rate; TpTend, T‐peak to T‐end interval duration; Tsym, T wave area symmetry ratio.

## 4 DISCUSSION

In the present study, we sought to determine whether individuals with T1D and IAH (diagnosed via Gold questionnaire) were protected against proarrhythmogenic effects of hypoglycaemia due to their assumed impaired sympathoadrenal responses. We showed that hypoglycaemia led to proarrhythmogenic electrocardiographic changes even in this group of high‐risk individuals and that these responses did not largely differ from electrocardiographic changes induced by hypoglycaemia in individuals with no proven IAH. One of the potential explanations for these findings could be offered in the study by Rubin et al., which reported that, at least in a proportion of individuals with T1D, Gold/Clarke questionnaires poorly correlated with adrenaline responses to experimental hypoglycaemia.^16^ A large proportion of individuals with possible or definite CAN (together 6/14, 43%) were included in this study. We have previously shown that in experimental settings, individuals with CAN, in comparison to those without CAN, exhibited abnormalities in response to adrenaline infusion including greater increases in heart rate or changes in T wave morphology at lower adrenaline concentrations, most likely due to the presence of denervation adrenergic hypersensitivity.^27^ To conclude, the character and magnitude of electrocardiographic responses to hypoglycaemia which were observed in this group of individuals with T1D and IAH are most likely a result of complex interplay between blunted sympathoadrenal responses, the degree of denervation adrenergic hypersensitivity, and likely other, in part unknown, mechanisms.

Higher magnitude of electrocardiographic changes was detected in response to daytime hypoglycaemia in comparison to nocturnal hypoglycaemia. It has been previously shown that sleep impairs counter‐regulatory responses to hypoglycaemia in both people with diabetes and healthy volunteers^28^ and that these responses are also affected by the body posture at which hypoglycaemia occurs.^29^ Diurnal differences in electrophysiological responses to hypoglycaemia were also noted in our previous observational studies.^8^, ^9^

In relation to cardiac arrhythmias, very few were detected and these were without clinical significance. This is not surprising because arrhythmias were monitored over a short period of time and in a small number of participants. In future studies, we suggest substantially longer periods of observation on a larger number of individuals to address this issue.

In this study, we detected, as expected, a high proportion of asymptomatic hypoglycaemic episodes both at night and during the day (>75%) and this proportion was higher than the one detected in the already discussed study on T1D individuals with largely intact awareness of hypoglycaemia, in which 49% of daytime and 76% of nocturnal hypoglycaemic episodes were asymptomatic.^9^

The following were the main strengths of this study. Electrocardiographic responses to hypoglycaemia were studied in comparison to person‐ and time‐matched euglycaemic episodes. Apart from the robustness of such a protocol, it enabled us to include individuals on QT‐interval prolonging medications because each participant served as their own control. Second, we provide detailed participant characteristics, including information on their insulin regimen, QT‐prolonging medication use, and the number of hypoglycaemic episodes each individual contributed to the analysis. Lastly, this study was undertaken at the time before the AHCL systems were introduced into clinical practice in the UK. These systems have shown impressive reductions in time spent in hypoglycaemia^30^, ^31^ and have become a standard of care for the high‐risk individual with T1D and IAH. Our study thus provides a valuable set of data because the execution of this study would be very difficult in the UK in the present time as more participants on AHCL systems would be required to take part in order to record a similar (or higher) number of hypoglycaemic episodes. Temporarily stopping the AHCL systems for the duration of the study would not be possible in this high‐risk group of individuals for ethical reasons; yet, more studies into the proarrhythmogenic potential of hypoglycaemia are required.

One of the main limitations of our study is the relatively low number of participants and the resulting low number of hypoglycaemic episodes that could be studied. The original study plan anticipated the recruitment of a larger number of participants, but the recruitment into the study had to be paused in March 2020 due to the outbreak of the Covid‐19 pandemic. Second, rapid advances in the field of diabetes technology, in particular the widespread use of AHCL systems in individuals with T1D and IAH, meant that once the Covid‐19 pandemic was over, it was not possible to recruit further participants into the study. Other limitations are a direct consequence of the observational nature of the study. This meant that no direct measurements of catecholamine or potassium levels at the time of hypoglycaemia were possible and that the clinical diagnosis of IAH was not accompanied by biochemical evidence of diminished sympathoadrenal response to hypoglycaemia obtained via a hyperinsulinaemic‐hypoglycaemic clamp study.

Given the results of the present study and the considerations discussed above, we conclude that further studies with a larger number of participants with T1D and IAH will be required in order to gain a nuanced understanding of the factors influencing cardiac repolarisation and the risk of cardiac arrhythmias during hypoglycaemia in this high‐risk group of individuals. The protocols of such studies should ideally include concomitant CGM and ECG monitoring over longer periods of time and detailed phenotyping of participants including genetic studies.

## AUTHOR CONTRIBUTIONS

A.I., S.R.H., and P.N. co‐conceived the study. A.I., P.N., A.B., J.L., and S.R.H. developed the study protocol and data collection tools. Data collection was performed by P.N., A.B., E.D., A.S., M.F.A., A.I.O., and A.I. Data analysis was performed by A.B., P.N., and R.M.J. The first draft of the manuscript was prepared by P.N. with critical input on multiple versions of the manuscript from all authors. All authors approved the final submitted version. A.I. and S.R.H. are the guarantors of the work and, as such, had full access to all study data and thus take responsibility for data integrity and accuracy of data analysis.

## FUNDING INFORMATION

This is a summary of independent research funded in part by the University of Sheffield Pump Priming Award (grant number X/004622‐24) awarded to P.N. and conducted at the National Institute for Health Research (NIHR) Sheffield Clinical Research Facility. The views expressed are those of the authors and not necessarily those of the University of Sheffield, NHS, the NIHR, or the Department of Health.

## CONFLICT OF INTEREST STATEMENT

P.N. has served on speaker panels for Novo Nordisk, Eli Lilly, Sanofi, Boehringer‐Ingelheim, Berlin‐Chemie Menarini, Mundipharma, Krka, Viatris, Novartis, Medtronic and Abbott; on advisory panels for Sanofi, Boehringer‐Ingelheim and Novartis; received honoraria or consulting fees from Merck, Boehringer‐Ingelheim and Eli Lilly; and received travel grants from Sanofi, Novo Nordisk, Eli Lilly, Viatris and Berlin‐Chemie Menarini. A.I. received speaker fees from Astra Zeneca, Eli Lilly and educational grant support from Sanofi and also received research support from Dexcom Inc. S.R.H. received research grants from Dexcom Inc. He has served on speaker panels for NovoNordisk for which he has received remuneration. He has served on advisory panels or as a consultant for Vertex, Zealand and Zucara for which his institution has received remuneration. All other authors of this work have no relevant conflict of interest to disclose.

## Supporting information


Table S1.

Table S2.

Table S3.

## Data Availability

The data that support the findings of this study are available from the corresponding author upon reasonable request.
