# Supplementary material for: Electrocardiograpic responses during spontaneous hypoglycaemia in people with type 1 diabetes and impaired awareness of hypoglycaemia
Source: Diabet Med. 2025 Feb 27;42(7):e70019. doi: 10.1111/dme.70019 (PMC12151816; doi:10.1111/dme.70019)
Supplement: Supplementary file 1 — Table S1. [file DME-42-e70019-s001.docx]

**Supplementary table 1.** Individual participant’s characteristics

| ID | Sex | Age  (years) | BMI  (kg/m^2^) | T1D duration  (years) | Gold score | HbA_1C_  (mmol/mol) | CAN  status | Insulin  regimen | CGM | Microvascular  complications | No. of  hypos* | No. of analysable  hypos** | QT-prolonging  medications |
| --- | --- | --- | --- | --- | --- | --- | --- | --- | --- | --- | --- | --- | --- |
| #1 | F | 27 | 29.6 | 24 | 6 | 67 | no | lispro/degludec | No | NPDR | 6 | 5 | lofepramine |
| #2 | F | 32 | 25.4 | 13 | 7 | 49 | no | lispro/detemir | No | NPDR | 5 | 0 | none |
| #3 | F | 39 | 21.6 | 28 | 5 | 89 | possible | lispro/degludec | No | NPDR | 3 | 3 | none |
| #4 | M | 26 | 22.0 | 23 | 4 | 62 | no | aspart/detemir | No | NPDR | 0 | 0 | none |
| #5 | F | 42 | 28.1 | 24 | 6 | 54 | possible | lispro via CSII (AccuChek Spirit Combo) | Dexcom  G6 | NPDR, DSPN,  CKD2 | 4 | 4 | amitriptyline |
| #6 | F | 29 | 28.7 | 12 | 4 | 62 | no | lispro/degludec | No | NPDR | 1 | 1 | none |
| #7 | F | 34 | 32.4 | 24 | 5 | 67 | no | lispro/degludec | No | NPDR | 1 | 1 | none |
| #8 | F | 44 | 29.4 | 34 | 4 | 72 | no | aspart/detemir | No | NPDR, CKD2 | 0 | 0 | none |
| #9 | F | 35 | 26.1 | 11 | 7 | 116 | definite | lispro/degludec | No | NPDR, DSPN | 0 | 0 | none |
| #10 | F | 30 | 20.1 | 20 | 5 | 120 | definite | aspart/degludec | No | PDR, CKD3 | 0 | 0 | none |
| #11 | M | 47 | 20.3 | 23 | 7 | 99 | definite | aspart/detemir | No | Renal transplant | 0 | 0 | tacrolimus |
| #12 | F | 48 | 26.2 | 42 | 5 | 58 | no | lispro via CSII (AccuChek Spirit Combo) | No | DSPN | 4 | 3 | pregabalin  venlafaxine |
| #13 | M | 55 | 22.0 | 21 | 6 | 83 | possible | aspart/degludec | No | NPDR | 4 | 2 | trazodone |
| #14 | M | 53 | 32.7 | 37 | 4 | 53 | ND | aspart/degludec | No | PDR | 7 | 7 | none |

Data are displayed as mean ± SD (range) or n/n (%). Abbreviations: BMI, body mass index; CAN, cardiovascular autonomic neuropathy; CGM, continuous glucose monitoring; CKD, chronic kidney disease; CSII, continuous subcutaneous insulin infusion; DSPN, distal symmetric polyneuropathy; NPDR, non-proliferative diabetic retinopathy; PDR, proliferative diabetic retinopathy; QTc, QT interval corrected for heart rate; T1D, type 1 diabetes; *number of all hypoglycaemic episodes experienced during the study period; ** number of hypoglycaemic episodes with available time and person matched euglycaemia that were used for electrophysiological and HRV analysis.
