# Supplementary material for: Electrocardiograpic responses during spontaneous hypoglycaemia in people with type 1 diabetes and impaired awareness of hypoglycaemia
Source: Diabet Med. 2025 Feb 27;42(7):e70019. doi: 10.1111/dme.70019 (PMC12151816; doi:10.1111/dme.70019)
Supplement: Supplementary file 2 — Table S2. [file DME-42-e70019-s003.docx]

**Supplementary table 2.** Electrocardiographic responses to episodes of hypoglycaemia and person and time matched euglycaemia

| ID | Time of  hypo episode | daytime / night time  hypo episode | Hypoglycaemia | | | Euglycaemia (person and time matched) | | |
| --- | --- | --- | --- | --- | --- | --- | --- | --- |
|  |  |  | **QTc (ms)** | **TpTend (ms)** | **Tsym** | **QTc (ms)** | **TpTend (ms)** | **Tsym** |
| #1 | 21:17 | daytime | 493.0 | 104 | 1.146 | 433.9 | 77 | 1.216 |
| #1 | 00:52 | night time | 451.7 | 85 | 1.080 | 451.3 | 80 | 1.443 |
| #1 | 19:47 | daytime | 513.8 | 122 | 0.996 | 450.3 | 88 | 1.113 |
| #1 | 00:02 | night time | 454.0 | 104 | 0.864 | 458.1 | 80 | 1.231 |
| #1 | 21:47 | daytime | 477.8 | 126 | 0.449 | 405.6 | 68 | 1.173 |
| #3 | 02:36 | night time | 414.9 | 75 | 1.597 | 397.9 | 62 | 1.636 |
| #3 | 00:55 | night time | 390.3 | 62 | 1.674 | 393.1 | 66 | 1.519 |
| #3 | 05:00 | night time | 391.0 | 62 | 1.643 | 391.5 | 63 | 1.612 |
| #5 | 20:59 | daytime | 430.8 | 64 | 1.761 | 475.4 | 81 | 1.468 |
| #5 | 02:24 | night time | 466.5 | 81 | 1.895 | 452.2 | 82 | 1.455 |
| #5 | 03:53 | night time | 431.5 | 71 | 1.670 | 434.6 | 68 | 1.720 |
| #5 | 22:28 | daytime | 423.3 | 72 | 1.342 | 422.3 | 63 | 1.659 |
| #6 | 14:43 | daytime | 420.0 | 86 | 1.057 | 423.2 | 81 | 1.382 |
| #7 | 20:58 | daytime | 458.9 | 109 | 1.518 | 427.3 | 89 | 1.390 |
| #12 | 00:13 | night time | 504.7 | 128 | 1.599 | 462.9 | 84 | 2.476 |
| #12 | 12:33 | daytime | 476.6 | 95 | 1.642 | 425.8 | 75 | 1.856 |
| #12 | 19:48 | daytime | 463.7 | 99 | 1.404 | 459.4 | 86 | 1.712 |
| #13 | 03:11 | night time | 403.3 | 66 | 1.763 | 415.3 | 79 | 1.738 |
| #13 | 09:11 | daytime | 412.8 | 73 | 1.612 | 402.3 | 66 | 1.533 |
| #14 | 02:12 | night time | 366.0 | 83 | 1.176 | 380.0 | 91 | 1.132 |
| #14 | 17:02 | daytime | 402.2 | 81 | 1.044 | 410.5 | 85 | 1.136 |
| #14 | 22:02 | daytime | 430.2 | 96 | 0.739 | 390.3 | 69 | 1.327 |
| #14 | 22:32 | daytime | 387.2 | 90 | 0.910 | 393.1 | 76 | 1.133 |
| #14 | 00:37 | night time | 426.1 | 102 | 0.834 | 401.9 | 95 | 1.019 |
| #14 | 21:52 | daytime | 412.5 | 87 | 1.014 | 385.7 | 67 | 1.298 |
| #14 | 02:42 | night time | 394.1 | 97 | 1.001 | 382.8 | 93 | 1.088 |

Abbreviations: ID, participant’s identification number; QTc, QT-interval corrected for heart rate; TpTend, T-peak to T-end interval duration; Tsym, T wave area symmetry ratio
