# Supplementary material for: Electrocardiograpic responses during spontaneous hypoglycaemia in people with type 1 diabetes and impaired awareness of hypoglycaemia
Source: Diabet Med. 2025 Feb 27;42(7):e70019. doi: 10.1111/dme.70019 (PMC12151816; doi:10.1111/dme.70019)
Supplement: Supplementary file 3 — Table S3. [file DME-42-e70019-s002.docx]

**Supplementary table 3.** Individual beat count of cardiac arrhythmias during hypoglycaemia and euglycaemia hours

| **Type of arrhythmia** | **Participant**  **ID** | **Hypoglycaemia** | | **Euglycaemia** | | **IRD** | **95% CI** |
| --- | --- | --- | --- | --- | --- | --- | --- |
|  |  | **count** | **hours** | **count** | **hours** |  |  |
| **AEBs** | #1 | 0 | 5 | 0 | 30 |  |  |
|  | #2 | 0 | 6 | 1 | 23 | -0.044 | -0.221 to 0.134 |
|  | #3 | 0 | 2 | 0 | 16 | 0 | 0 |
|  | #4 | 0 | 0 | 0 | 30 |  |  |
|  | #5 | 0 | 1 | 0 | 50 | 0 | 0 |
|  | #6 | 0 | 0 | 3 | 40 |  |  |
|  | #7 | 0 | 3 | 0 | 21 | 0 | 0 |
|  | #8 | 0 | 0 | 13 | 48 |  |  |
|  | #9 | 0 | 0 | 1 | 24 |  |  |
|  | #10 | 0 | 0 | 0 | 2 |  |  |
|  | #11 | 0 | 0 | 1 | 2 |  |  |
|  | #12 | 1 | 4 | 16 | 52 | -0.058 | -0.777 to 0.662 |
|  | #13 | 7 | 1 | 73 | 24 | 3.958 | -10.529 to 18.446 |
|  | #14 | 1410 | 10 | 10912 | 58 | -47.138 | -234.612 to 140.336 |
| **single VPBs** | #1 | 0 | 5 | 1 | 30 | -0.333 | -0.202 to 0.135 |
|  | #2 | 0 | 6 | 0 | 23 | 0 | 0 |
|  | #3 | 0 | 2 | 1 | 16 | -0.063 | -0.477 to 0.322 |
|  | #4 | 0 | 0 | 1 | 30 |  |  |
|  | #5 | 0 | 1 | 4 | 50 | -0.080 | -0.636 to 0.476 |
|  | #6 | 0 | 0 | 1 | 40 |  |  |
|  | #7 | 0 | 3 | 0 | 21 | 0 | 0 |
|  | #8 | 0 | 0 | 1291 | 48 |  |  |
|  | #9 | 0 | 0 | 1 | 24 |  |  |
|  | #10 | 0 | 0 | 0 | 2 |  |  |
|  | #11 | 0 | 0 | 1 | 2 |  |  |
|  | #12 | 0 | 4 | 11 | 52 | -0.212 | -0.886 to 0.463 |
|  | #13 | 0 | 1 | 3 | 24 | -0.125 | -0.838 to 0.588 |
|  | #14 | 42 | 10 | 152 | 58 | 1.579 | -0.172 to 3.331 |

No other arrhythmias apart from those listed in the table were detected during hypoglycaemia and are therefore not reported. Abbreviations: AEBs, atrial ectopic beats; IRD, incidence rate difference; VPBs, ventricular premature beats.
